# Effect of remelting heat treatment on the microstructure and mechanical properties of SnBi solder under high-speed self-propagation reaction

**DOI:** 10.1038/s41598-022-13776-z

**Published:** 2022-06-09

**Authors:** Yang Wan, Longzao Zhou, Fengshun Wu

**Affiliations:** 1 School of Materials Science and Engineering, Huazhong University of Science and Technology, Wuhan, 430074 China; 2 TKD Science and Technology Co., LTD, Suizhou, 441300 China

**Keywords:** Metals and alloys, Characterization and analytical techniques, Electrical and electronic engineering

## Abstract

The heat source based on the self-propagation reaction of Al/Ni thin foil has the characteristics of concentrated heat, fast temperature rise/fall rate and small heat-affected zone; it can complete the melting and solidification crystallization of solder within milliseconds to realize solder interconnection, which can solve the problems of damage to heat-sensitive materials and components caused by monolithic heating of package structure. However, due to the highly non-stationary interconnection process, the resulting microstructure morphology may affect the service performance of the interconnected joints. In view of this, to investigate the post-solder microstructure of solder based on the self-propagation reaction, this paper analyzes the effect of the initial microstructure on the post-solder microstructure by heating 300-μm-thick SnBi solder with a 40-μm Al/Ni thin foil. The results indicated that the short melting time could resulted in the incomplete melting of heterogeneous phases and the non-uniform distribution of elements during the melting process, which had a significant effect on the morphology and composition distribution of the solidified microstructure, as well as the hardness distribution of the melted zone. The above conclusions have the potential to improve the interconnection process based on the self-propagation reaction, which is critical for both theoretical guidance and engineering application.

## Introduction

Electronic packaging solder interconnection processes are typically accomplished through the device’s integral heating. Due to the material's different coefficients of thermal expansion (CTE), a concentration of thermal stress forms at the interface, causing damage to the device's internal thermal sensitivity, thermal mismatch components and materials, and decreased package reliability. Since SnPb was banned in electronic information products because of its inherent toxicity, Sn-based lead-free solders have been widely studied and commercially used to replace Sn–Pb solders. In recent years, the demand for lead-free solder continues to grow, and many Pb-free solders have been studied. Yuanyuan Qiao et al.^1^ used Quasi- in-situ method to observe the growth behavior of intermetallic compounds (IMCs) in Cu/Sn-3.0Ag-0.5Cu/Cu micro solder joints with single β-Sn grain during aging with and without temperature gradient (TG), and come up with a solution to predict the IMC morphology and thickness considering β-Sn grain orientation. Xiaoyang Bi et al.^2^ found that the addition of Co–Ni film enhanced the mechanical properties of Ni film and Ni3Sn4 IMC. Haozhong Wang et al.^3^ confirmed through tests that the hardness and modulus of Sn-3.0Ag-0.5Cu composite solder alloys were enhanced after the addition of Ni-CNTs. The above literature are from the perspective of adding elements to study how to improve the strength of the solder after the conventional soldering method, but less research on how to improve the soldering strength in the high-speed self-propagation reaction, in view of this, by studying the effect of remelting heat treatment on the microstructure and mechanical properties of SnBi solder under high-speed self-propagation reaction, this paper is critical for both theoretical guidance and engineering application.

The self-propagation reaction interconnection technology is capable of resolving the aforementioned issues more effectively. Self-propagation reaction solder interconnection technology melts the solder by utilizing the self-heating and self-conducting effect of the high chemical reaction heat between reactants. Due to their ease of excitation and high thermal efficiency, nano-thin foils with alternating Al and Ni nano-layers, commonly referred to as AlNi self-propagation thin foils, are one of the most common self-propagation reaction materials used in package interconnects. The reaction equation is denoted by the symbol Eq. (1).

1 $$\begin{array}{*{20}c} { \hbox{Al} + \hbox{Ni} \to \hbox{NiAl} + 129.2{\text{ kj}}/{\text{mol}}} \\ \end{array}$$

Heerden et al.^4^ completed the interconnection between silicon chips and copper heat sinks using SnPb solder and AlNi self-propagating thin foil reaction, and applied the technique to the rework of failed devices; Qiu et al.^5^ used AlNi self-propagating thin foil reaction to achieve direct bonding between silicon wafers and passed the IPA leakage test; Namazu et al.^6^ used magnetron sputtering to deposit thin AlNi foil atop AuSn solder and then exploited the heat generated by the thin foil's ignition to melt the solder to connect MEMS devices; Levin et al.^7^ used self-propagation to exothermally melt AuSn solder at room temperature in order to complete the connection between electrical connectors and printed circuit boards.

To enable device connectivity, these applications used a thin foil self-propagation reaction that was exothermic to melt the solder. However, due to the fast self-propagation reaction and highly concentrated heat source, the solder rapidly warms/cools (approximately 105~10^7^ °C/s) due to the very short period (about 0.2 ms), resulting in a very large temperature gradient in the solder layer (1~3*10^7^ °C/m)^8–10^. During the melting process, the solder that has been impacted by the short liquid state period cannot be thoroughly convection mixed. The elemental diffusion is also affected by the short liquid time leading to the crystalline microstructure of the solder solidification that is different from the conventional melting crystalline microstructure, which shows a specific inheritance of the original microstructure of the solder^11–13^. Due to the scarcity of studies on this subject, this paper examines the effect of the solder's initial microstructure on the microstructure of the joint’s melting zone under the action of a self-propagating high-speed heat source^14–16^.

## Materials and methods

### Materials

**Figure 1:**
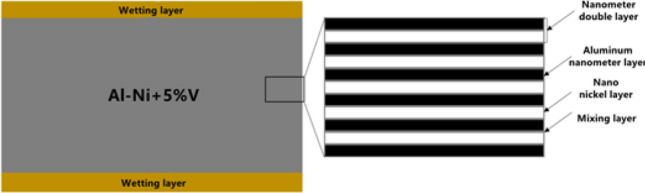
Structure of AlNi nano-thin foil.

The self-propagation reaction described in this article is based on an Indium nanomultilayer foil (NanoFoil). The thin foil is an Al–Ni reactive multilayer foil with the structure depicted in Fig. 1. The Al/Ni nano foil has a thickness of 40 μm and an atomic ratio of Al to Ni of 1:1, resulting in AlNi as the end product. On the surface of the foil, a wetting layer (59 wt% Ag~27.25 wt% Cu~12.5 wt% In~1.25 wt% Ti) with a thickness of 1 μm is coated to improve the wetting between the self-propagating foil and the solder layer.

**Figure 2:**
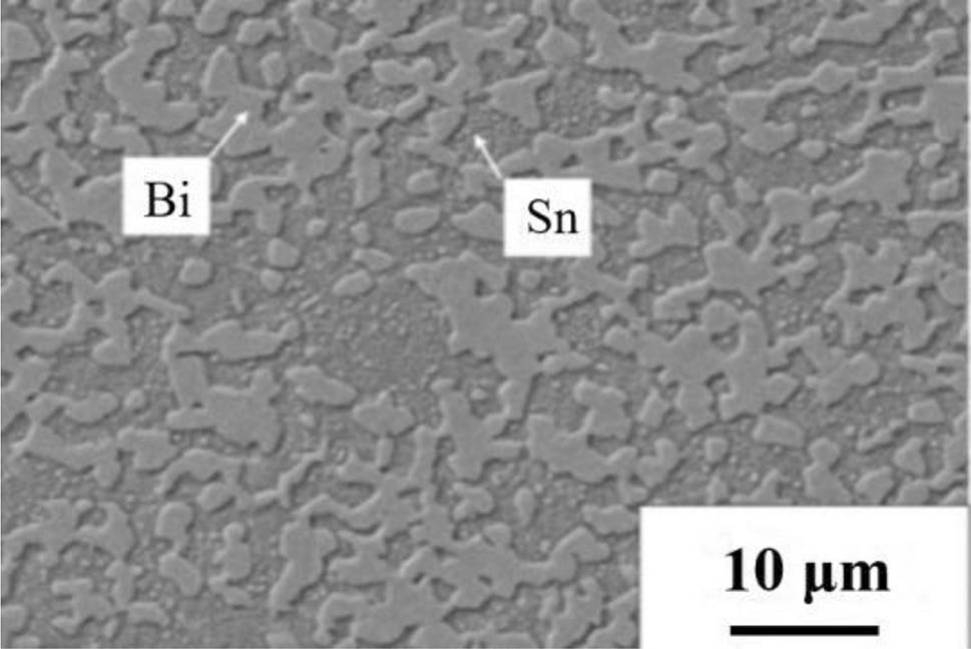
SnBi solder sheet microstructure morphology.

The prefabricated solder sheets used in this paper were provided by Shaanxi Turing Company. The thick (300 μm) SnBi solder sheets were selected for self-propagation melting to partially melt the solder sheets and allow intuitive observation of the difference between the original solder microstructure and the re-solidified microstructure under the action of the self-propagation high-speed heat source. Figure 2 depicts the microstructure of these solder sheets.

### Equipment

**Figure 3:**
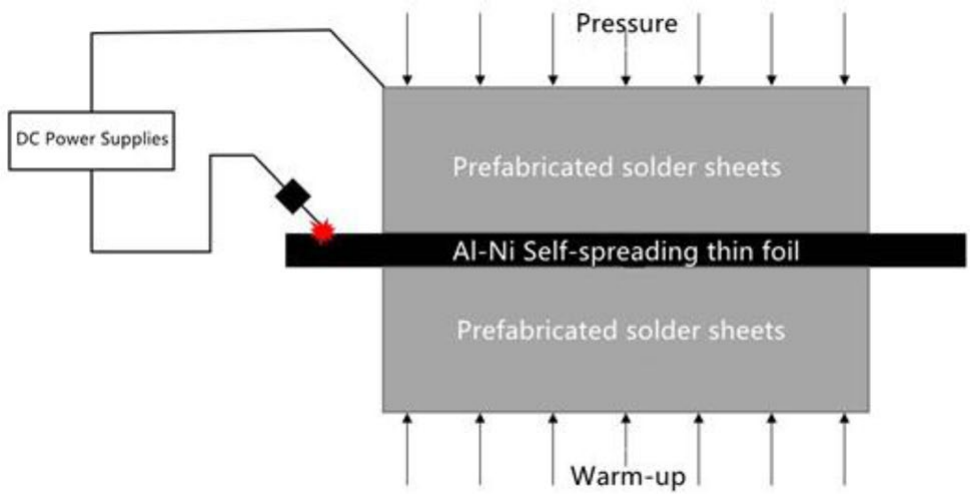
Schematic diagram of solder/self-propagating thin foil/solder sandwich structure.

The experimental design utilized in this article is depicted in Fig. 3. To study the non-equilibrium microstructure distribution throughout the solder's melting zone when subjected to a high-speed heat source, a sandwich structure of solder/self-propagating foil/solder was used. Additionally, varied pressures and preheating temperatures were applied to the reaction structure during the reaction to ensure that the solder and self-propagating foil fit tightly and formed a reliable connectivity. When the preheating temperature exceeds the Al/Ni foil’s ignition point (~ 200 °C), the Al/Ni foil ignites directly, and the long preheating period accelerates interdiffusion between the Al and Ni nano-layers, reducing the total heat yield and heat generation efficiency.

## Methods

**Figure 4:**
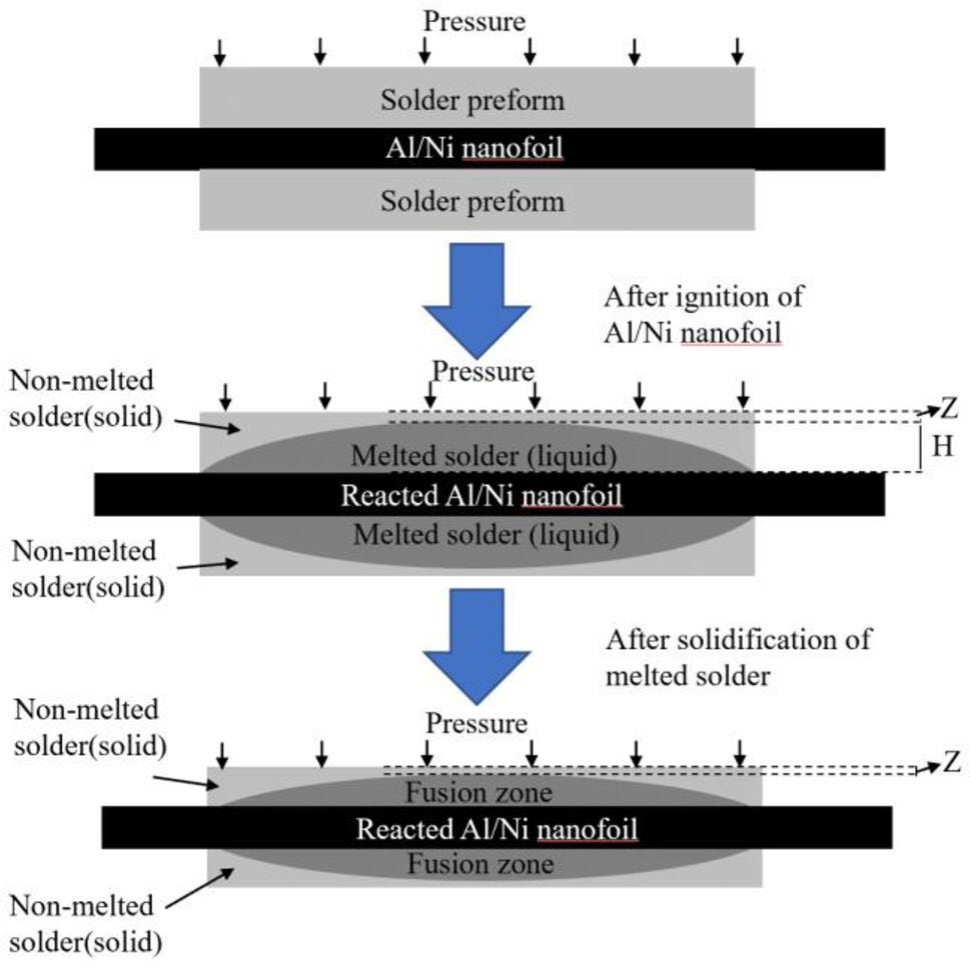
Schematic diagram of the experimental process.

The SnBi solder sheets were separated into two groups. The solder sheets in group A were left untreated, while those in group B were melted and cooled to room temperature at a rate of 10 °C/s. Then, for each group, a self-propagating foil was inserted between the two components of the prefabricated solder. After applying pressure to this sandwich construction, the self-propagating foil reacted with energy excitation, generating sufficient heat to melt the two groups of solder sheets on both sides and produce a joint, as illustrated in Fig. 4.

## Results

### Interfacial microstructure of solder joints

**Figure 5:**
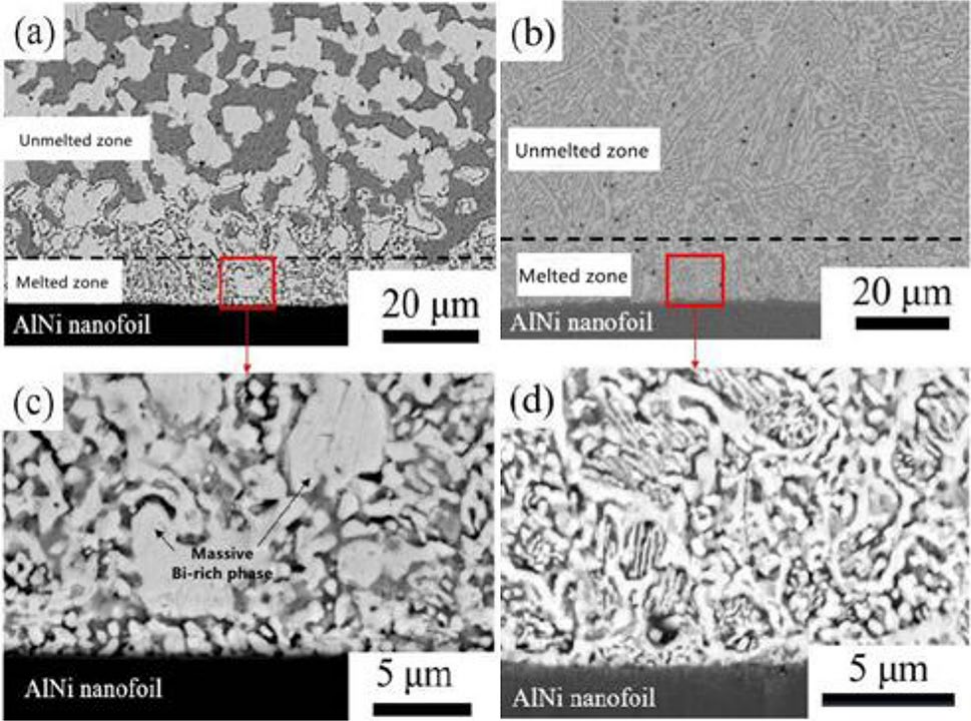
The complete microstructure of SnBi solder and the microstructure in the melting zone. (**a**, **c**) unheated SnBi solder, (**b**, **d**) heat-treated SnBi solder.

Both groups' solders were heated by using a self-propagating heat source, and the resulting microstructures were compared. The results are illustrated in Fig. 5. Each group's solidification microstructures were distinct under the influence of the identical self-propagating high-speed heat source. After the self-propagating high-speed heat source reacted on the solders of group A, the microstructure of the melting zone retained a significant residual Bi-rich phase with a high melting point that was similar to the initial microstructure. In the case of solders in group B, the melting zone microstructure exhibited a laminar eutectic microstructure that was also similar to the initial microstructure of the remelted SnBi eutectic solder. It was established that the initial microstructure of the SnBi solder had a significant effect on the microstructure morphology after the self-propagating high-speed heat source was applied.

**Figure 6:**
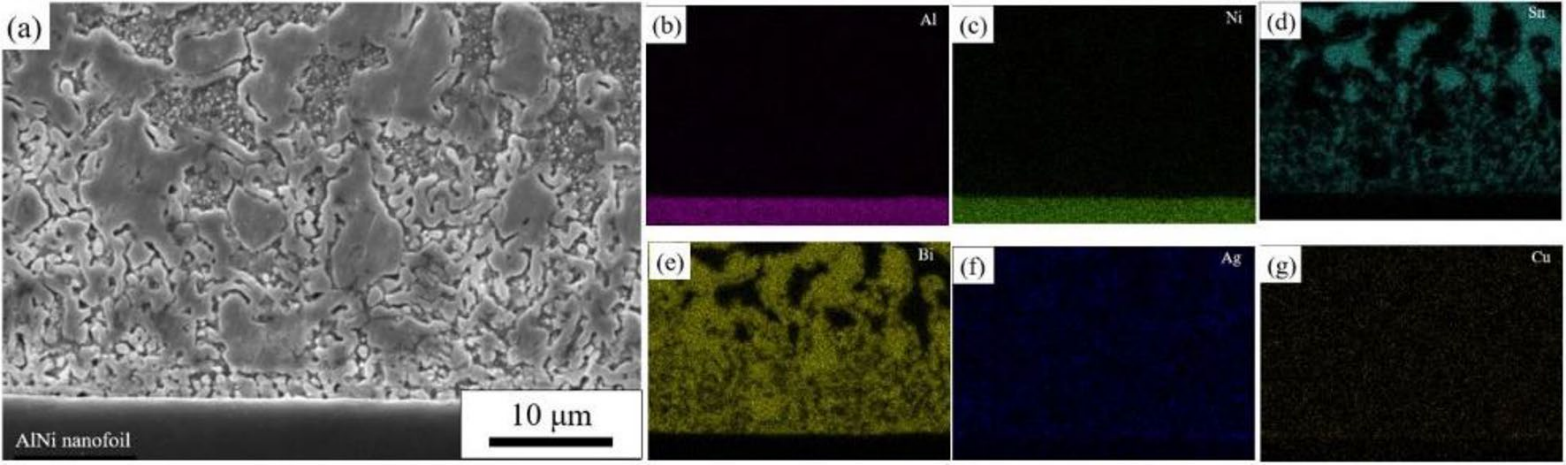
Microstructure and surface scan results of the melting zone of SnBi solder after the action of self-propagating high-speed heat source.

By scanning the surface of the SnBi solder in group A, it was found that the AlNi thin foil formed an obvious interface with the solder and exhibited no evidence of mutual diffusion, as shown in Fig. 6. The unmelted region’s Sn- and Bi-rich phases were uniformly distributed and of uniform size. Concerning the melted region, the size distribution of the Bi-rich phase was not uniform; the highest size reached 5 μm, while the smallest size was less than 1 μm. These large residual phases form in a manner that is clearly distinct from that of other fine eutectic structures. And the primary mechanism of formation was a scarcity of element dispersion.

### Interfacial composition distribution of solder joints

**Figure 7:**
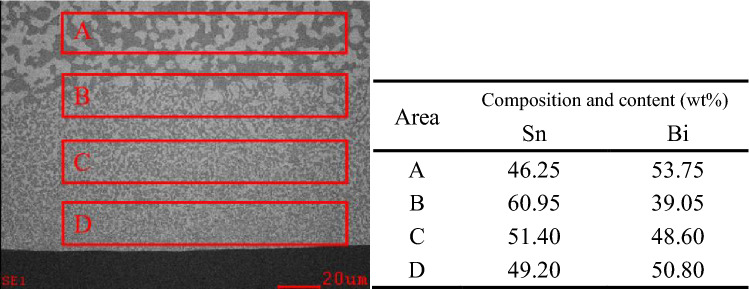
Composition of SnBi solder in different regions after the action of self-propagating high-speed heat source.

The SnBi composition of the entire melting zone diverged from the eutectic point (Sn 42%–Bi 58%), but the melting zone retained an alternating eutectic pattern of Sn-rich and Bi-rich phases. The EDX composition analysis indicated a distinct gradient distribution of Sn content in the melting zone, with a lower Sn content towards the self-propagating thin foil's surface and a higher Sn content away from it. This was due to the melting temperatures of the SnBi solder’s Sn-rich and Bi-rich phases being different. Due to the higher melting point, the Bi-rich phase was more difficult to melt than the Sn-rich phase during the melting process, which resulted in the Sn content in the melting zone was higher than the eutectic point at the beginning of melting. Meanwhile, the solder solidified in a direction away from the melting zone's interface with the unmelted region and toward the melting zone’s interface with the self-propagating thin foil. When combined with Fig. 7, the composition of SnBi solder in various regions following the operation of a self-propagating high-speed heat source was clearly deduced. The composition of the melt during the initial solidification process was subeutectic, and the SnBi solder was prone to the formation of the Sn primary phase. The solder solidified in a very non-equilibrium state, the cooling rate and supercooling degree were both high, and the eutectic precipitation occurred below the eutectic point, indicating that the alloy liquid was supersaturated for both the Sn and Bi phases. Both Sn and Bi crystallized during crystallization, and the eutectic microstructure was nevertheless precipitated when the composition diverged from the eutectic point, resulting in the formation of a pseudo-eutectic microstructure (Sn content slightly higher than the melt). Rapid solidification enclosed the incipient -Sn and prevented it from propagating to the self-propagating thin foil side, resulting in a high Sn concentration at the interface between the melted and unmelted areas at the start of solidification. The melt’s Sn concentration rapidly dropped as pseudoeutectic microstructure and incipient -Sn precipitated. As a result, the Sn content showed a gradient distribution that increased with distance from the self-propagating thin foil, and the closer the solder composition was to the eutectic point, the higher the Sn content.

**Figure 8:**
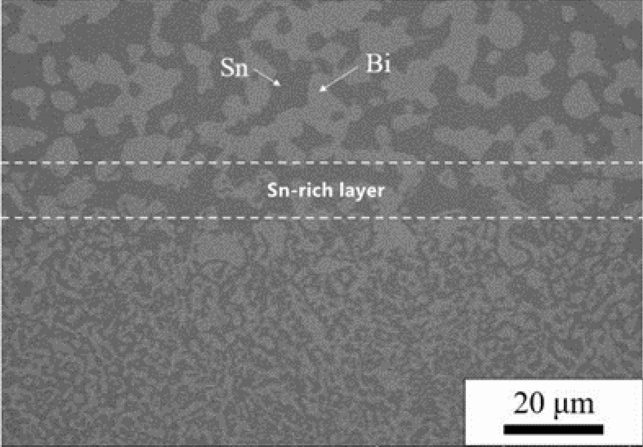
Microstructure of SnBi solder melt region/unmelted region interface.

The microstructure of the contact between the unmelted and melted regions of the SnBi solder was shown in Fig. 8. The Bi-rich phase in the unmelted region was found to have a distinct contour and a smooth interface with the Sn-rich phase. Within the paste region, a layer of Sn-rich phase encircled the big granular Bi-rich phase, followed by a layer of Bi-rich phase wrapped around the Sn-rich phase to form a eutectic structure. In the melting zone, the Bi-rich phase was alternatively dispersed with the Sn-rich phase. There was a Sn-rich layer at the interface between the unmelted and melted regions, and elemental analysis revealed that the Sn-rich layer had the same composition as the Sn-rich phase in the unmelted region, with a Sn content of 86.31–87.68%, and the solid solubility of Bi elements was higher than the equilibrium solid solubility (21%). The bulk Bi-rich phase that remained at this interface was elementally determined to be congruent with the equilibrium phase diagram description of the phase from which point-like Sn-rich particles precipitated.

**Figure 9:**
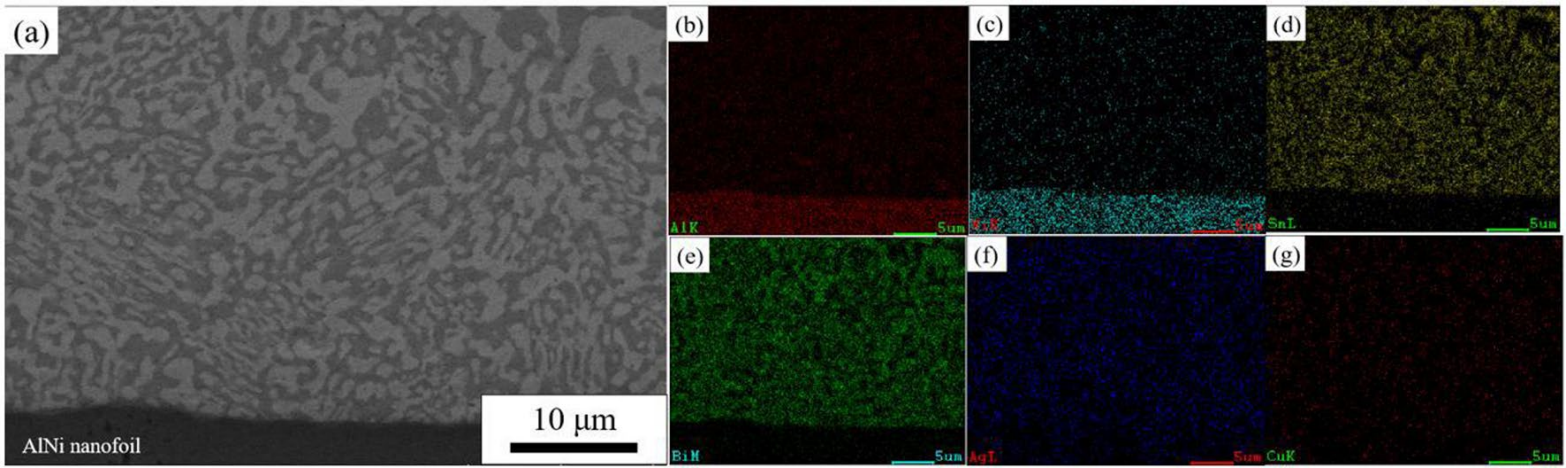
Microstructure and face scan element distribution in the melting zone of SnBi solder after heat treatment after the action of a self-propagating high-speed heat source. (**a**) Microstructure, (**b**) Al, (**c**) Ni, (**d**) Sn, (**e**) Bi, (**f**) Ag, (**g**) Cu.

Figure 9 illustrates further surface scans of remelted SnBi solder. The interface between the thin AlNi foil and the solder was readily apparent, with no obvious evidence of reciprocal diffusion. The size of the microstructure in the unmelted region was slightly larger than that in the melted region, and the Sn-rich and Bi-rich phases were smaller and more uniformly distributed than in the unremelted SnBi solder. The grain size of the Bi-rich phase was essentially less than 1 μm.

**Figure 10:**
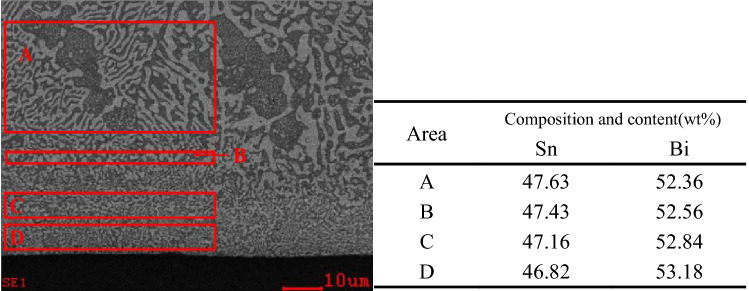
Composition of different regions of the heat-treated SnBi solder after the action of a self-propagating high-speed heat source.

Additionally, the elemental composition of the remelted SnBi solder was analyzed in each region. The outcome is depicted in Fig. 10. The elemental composition of Bi in the unmelted region was 52.36 wt%, and the microstructure morphology was also consistent with a sub-eutectic microstructure, with visible incipient –Sn phase generation and white Bi-rich phase particles precipitated in solid solution, indicating that the melt was sub-eutectic (58 wt%). At the interface between the melted and unmelted zones, the weight ratio of Sn to Bi was 47.43:52.56, which was still subeutectic. It was discovered that during remelting of SnBi solder, no Sn-rich layer formed at the melted/unmelted interface due to the action of the self-propagating high-speed heat source, and that the composition of this layer was essentially identical to that of the original microstructure. This occurred because, due to the tiny grain size and homogenous microstructure distribution of the remelted SnBi solder, both eutectic phases were consumed during the melting process, leaving no residual Bi-rich phases. And the melt was homogeneous in composition, being closer to the eutectic point than the unremelted SnBi solder melt composition^17,18^. As a result, no β-Sn phase could precipitate from the melted/unmelted interface to the self-propagating thin foil/solder contact during the solidification process, resulting in the absence of a Sn-rich band at the melted/unmelted solder interface. The composition was essentially uniform throughout the melting zone, with a slight gradient in the distribution of the Sn concentration. The gradient in the solidification composition was compatible with non-equilibrium solidification theory^19–21^, and the melt composition steadily approached the eutectic point as it solidified from the melted/unmelted interface to the solder/self-propagating thin foil Boundaries.

### Hardness distribution of solder joints

**Figure 11:**
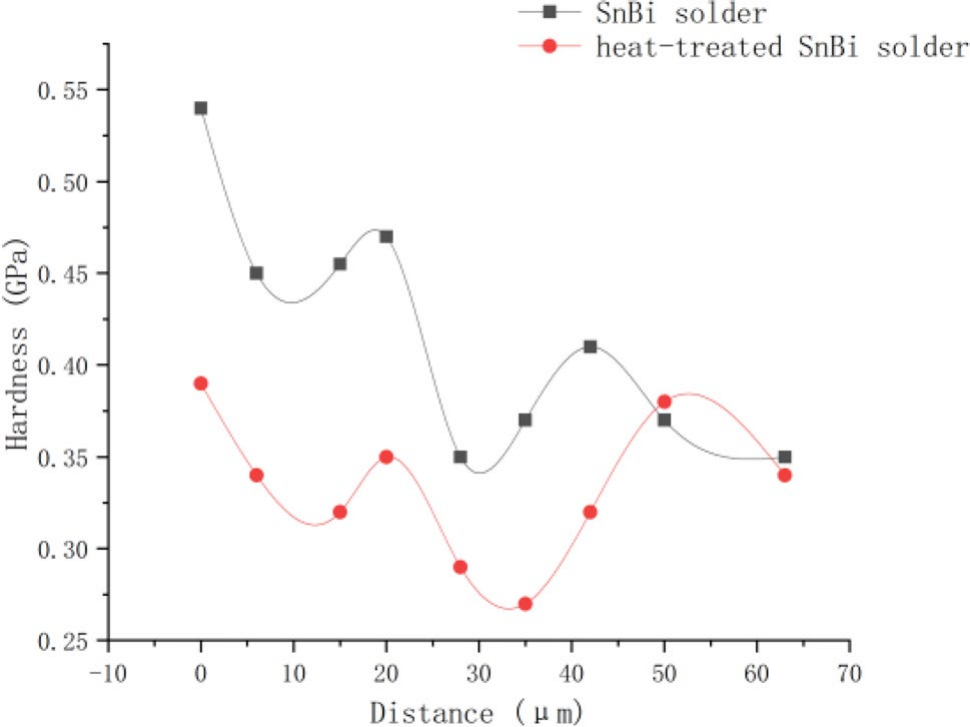
Hardness distribution of the microstructure surrounding the solder joint's melting interface.

Figure 11 illustrates the hardness distribution of the microstructure surrounding the solder joint's melting interface. Within the melting zone, the hardness of the SnBi solder rapidly decreases with increasing distance from the self-propagating foil, reaching 0.54 GPa on the side closest to the self-propagating foil and 0.35 GPa at a distance of 63 μm. This is because the grain size of the SnBi eutectic solder increases with distance from the self-propagating thin foil, and also the microstructure hardness in the melting zone increases with distance from the self-propagating thin foil. By comparing the microstructure hardness distributions within the melting zones of the two groups of solders, it was determined that the hardness of the heat-treated and refined SnBi solder was slightly less than that of the SnBi solder after the action of the self-propagating high-speed heat source, with a hardness distribution of 0.3–0.6 GPa for the non-heat-treated SnBi solder and 0.3–0.45 GPa for the heat-treated solder. SnBi solder that has been heat treated has a lower variance and a more concentrated hardness distribution. The statistical analysis of the unmelted zone’s hardness distribution reveals that the high hardness phase is mostly driven by the presence of a large Bi-rich hard and brittle phase. The solder which after heat-treatment is predominantly lamellar eutectic, and the SnBi solder is finer and lacks a substantial Bi-rich phase. Because the brittle and hard Bi-rich phase is essentially eliminated, the remelted solder’s hardness distribution is more uniform than that of unremelted SnBi solder. This indicates that as grain refinement improves, the average hardness of the solder falls, and the hardness distribution within the solder becomes more uniform.

## Discussion

According to prior research, the microstructure homogeneity of SnBi solder under the influence of a self-propagating high-speed heat source had a greater effect on the solidification microstructure morphology. Due to the solder's extremely rapid melting time under the influence of the self-propagating heat source, insufficient element diffusion may occur. Due to the inhomogeneous microstructure of the internal element diffusion, solders with a large size high melting point phase could not reach the eutectic point after a brief period of attaining the eutectic temperature^22,23^. This meant that the solder could not entirely melt, even when the region of the inhomogeneous microstructure of the solder reached the eutectic solder's melting point. Additionally, due to insufficient element diffusion, the region's original composition distribution would persist, preserving the region's original microstructure even if the temperature surpassed the melting point of the high melting point phase.

To further solve for the actual mass transfer process in the solder, the diffusion distance of the solute was typically computed using irregular atomic jumps^24^. To begin, it was previously established that the atomic jumping probability P, the jumping frequency, the jumping distance r, and the diffusion coefficient D are related as follows:

2 $$\begin{array}{*{20}c} {D = Pr^{2} \Gamma } \\ \end{array}$$

Suppose an atom made n-time jumps and represented each jump as a vector *r*, with the final jump vector *R*_*n*_

3 $$\begin{array}{*{20}c} {R_{n} = \sum r_{i} } \\ \end{array}$$

To find the modulus of *R*_*n*_, multiplied it by a dot product to obtain

4 $$\begin{array}{*{20}c} {{\text{R}}_{{\text{n}}}^{2} = {\Sigma }_{{{\text{i}} = 1}}^{{\text{n}}} {\text{r}}_{{\text{i}}}^{2} + 2{\Sigma }_{{{\text{j}} = 1}}^{{{\text{n}} - 1}} {\Sigma }_{{{\text{i}} = 1}}^{{{\text{n}} - {\text{j}}}} {\text{r}}_{{\text{i}}} \cdot {\text{r}}_{{{\text{i}} + {\text{j}}}} } \\ \end{array}$$

The leap of an atom was random, and the direction of each leap was independent of the previous leap, so the leap had the same probability for any vector direction, and any vector had a corresponding vector in the opposite direction, so the average value of the jump vector after a large number of atomic leaps $\overline{{R_{n}^{2} }}$ was expressed as

5 $$\begin{array}{*{20}c} {\overline{{R_{n}^{2} }} = nr^{2} } \\ \end{array}$$

Combining the atomic hopping probability P, hopping frequency Γ, hopping distance r, and the diffusion coefficient D in Eq. (2), the expression for the diffusion distance d could be obtained as

6 $$\begin{array}{*{20}c} {d = \sqrt {2atD} } \\ \end{array}$$

where a was the diffusion dimension.

Since the size of the heterogeneous phase Bi in the SnBi solder was about 10 μm, By calculating the diffusion distance, it was found that assuming the heterogeneous elements above the eutectic melting point which diffused to part of the solder had melted the diffusion coefficient and the time required to melt the heterogeneous phase of the SnBi solder were shown in Table 1^25^.

**Table 1:** Solute diffusion coefficient and melting time of the solder.

Materials	Heterogeneous phase size/μm	Diffusion coefficient 10^−9^/m^2^s^−1^	Melting time required/ms
SnBi	10	3.6	− 6.94

**Figure 12:**
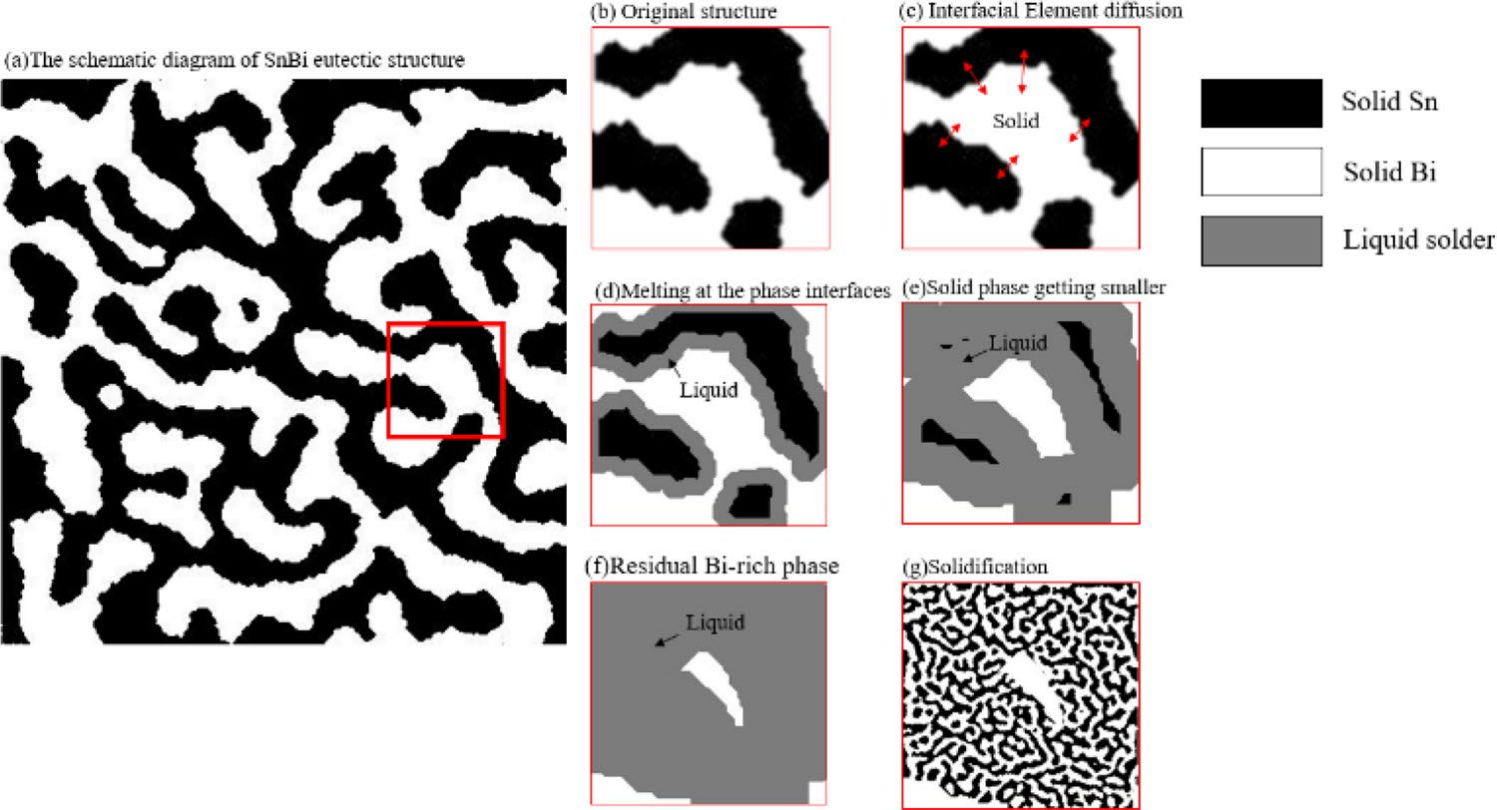
Schematic diagram of the melting process in the pasting region of SnBi solder.^26^

The microstructure formation process of SnBi eutectic solder with an uneven composition distribution was illustrated in Fig. 12 under the influence of a self-propagating high-speed temperature field^26–31^. The original microstructure was shown in Fig. 12a–b. When the initial microstructure was a relatively bulky eutectic microstructure before the temperature reached the eutectic point, the eutectic microstructure remained solid with irregular layer distribution. The phase interface between the Sn-rich phase and the Bi-rich phase was obvious, and there was a small interdiffusion of atoms between these two interfaces in the solid state. The microstructure had an obvious uneven distribution of components, and the melting point of the Sn-rich phase and Bi-rich phase would be higher than the eutectic point if these two phases were too late to diffuse. During the actual melting process, the atomic diffusion between the interfaces made the composition at the interface close to the eutectic composition, the melting point dropped to the eutectic point, and melting would start from the interface of the two phases as shown in Fig. 12b–e. With the extension of time, the volume of the liquid phase gradually increased, and both the Sn-rich phase and the Bi-rich phase were consumed. With the end of the self-propagation reaction, the temperature of the structure gradually decreased. The Sn-rich phase and the Bi-rich phase were continuously consumed before reaching the eutectic point, and the consumption would have not been stopped until the temperature reached the eutectic point, as shown in Fig. 12f. The solidification rate of the solder under the action of the self-propagating high-speed heat source was extremely high (200 mm/s), and the solidified microstructure was transformed into a finer layered eutectic than the original microstructure. The Bi-rich phase in the original microstructure was not completely consumed and remained in the newly formed microstructure as a lumpy distribution, as shown in Fig. 12g. There was obviously a layer of Sn-rich phase microstructure generated around the unmelted Bi-rich phase during solidification, which was because that this microstructure took the Bi-rich phase as the matrix during solidification, and the enrichment of Bi elements at this solid–liquid interface made it possible for the nucleation of the Bi-rich solid solution, and Bi would discharge the excess Sn-rich phase during the nucleation process, so there was a layer of wrapped Sn-rich phase around the unmelted Bi-rich phase, while the Sn-rich phase was also surrounded by a layer of wrapped Bi-rich phase. Taking the uneven distribution of the two phases in SnBi solder as a premise, if the solder temperature reached the melting point of Bi elements, i.e., above 271 °C, the solder would completely be melted at the moment, and the element diffusion rate in the melt would be fast as well, so this region would be more likely to present the eutectic state; if the solder temperature was above the Sn melting point and below the Bi melting point (231 °C < T < 271 °C), when the solder melting time was sufficient, both Sn monomers and Bi monomers could be completely melted. If the solder melting time was too short, incomplete melting of the residual phase could easily occurred.

Under the action of a self-propagating high-speed heat source, the time above the eutectic melting point temperature near the melted/unmelted interface in SnBi solder was mostly about 4–5 ms, and the maximum melting grain size would be 7.59–8.48 μm obtained by Eq. (6). Therefore, even at a distance of 10 μm from the melted/unmelted interface, it was not possible to completely melt the larger phases of the SnBi solder with uneven element distribution. The grain size produced by the solidified crystalline phase of the fully melted solder was about 1 μm, and most of the residual bulk phase was much larger than 1 μm, thus the grain size in the SnBi solder depended to a large extent on the particle size of the bulk microstructure. The farther the distance from the self-propagating foil, the shorter the melting time of the solder and the larger the lumpy microstructure, so the size of the microstructure in the SnBi solder would be increased with the increasing distance from the self-propagating foil. Insufficient melting was the mechanism for the formation of lumpy microstructure at the interface between the melted and unmelted regions^32^.

Compared to the lumpy microstructure that appeared at the interface between the melted and unmelted regions, granular lumpy microstructure also appeared near the self-propagating thin foil, and the size increased with increasing distance from the self-propagating thin foil. However, due to the short melting time of the solder, when the solder reached the heterogeneous phase melting point, the heterogeneous phase had been fully melted, the insufficient diffusion of the elements would still cause the residual solder in the completely melted region, forming a lumpy residual phase. Assuming that the convection effect of the melt had not been considered, the diffusion range of its heterogeneous elements was calculated by Eq. (6) and the melting time. It was clear to found that the element diffusion distance was smaller than the maximum heterogeneous phase size after complete melting of the solder with large original microstructure, and the liquid solder would still be distributed with Bi-rich and Sn-rich regions. During solidification, these regions had a higher melting point than the eutectic melt and solidify preferentially, so they could retain some of the characteristics of the original microstructure^33^. This led to the fact that even if the solder was completely melted, the uniformity of the initial microstructure distribution still influenced the composition and morphology of the solidified solder microstructure in the self-propagation reaction. The formation of residual phases close to the region of the self-propagating thin foil was owing to the appearance of lumpy microstructure caused by insufficient diffusion of the composition.

In summary, the incomplete melting of heterogeneous phases and the uneven distribution of elements during the solder melting process led to the formation of lumpy residual phases in the melting zone of the solder. The incompletely melted lumps were mainly concentrated at the melted/unmelted interface, while the smaller size lumps in the melting zone were formed by the uneven distribution of elements in the melt.

## Conclusion

The microstructure and distribution of low-temperature eutectic SnBi solder were compared in this article using two sets of double-sided SnBi solder sheets with and without remelt heat treatment using a 40 μm self-propagating thin foil. As a result, the following findings were reached:

On the one hand, the inhomogeneous initial microstructure distribution of Sn_42_Bi_58_ solder would have a significant impact on the morphology and composition distribution of the solidified microstructure under the action of the self-propagating high-speed heat source due to the short melting time; on the other hand, insufficient element diffusion during the solder melting process results in incomplete melting of heterogeneous phases and uneven distribution, which has a considerable effect on the composition and hardness distribution of the melting zone. According to the findings of this research, remelting SnBi solder prior to self-propagation high-speed soldering improves the consistency of microstructure morphology, composition, and hardness distribution in the melting zone after soldering, hence increasing the reliability of the soldering performance.

## Abbreviations

CTE: Coefficients of thermal expansion
IMC: Intermetallic compound
TG: Temperature gradient
CNTs: Carbon nanotubes
IPA: Isopropyl alcohol
MEMS: Micro-electro-mechanical system
